# Apigenin promotes apoptosis, inhibits invasion and induces cell cycle arrest of T24 human bladder cancer cells

**DOI:** 10.1186/1475-2867-13-54

**Published:** 2013-06-01

**Authors:** Yi Zhu, Yeqing Mao, Hong Chen, Yiwei Lin, Zhenghui Hu, Jian Wu, Xin Xu, Xianglai Xu, Jie Qin, Liping Xie

**Affiliations:** 1 Department of Urology, The First Affiliated Hospital, School of Medicine, Zhejiang University, 79 Qing Chun Road, Hangzhou, Zhejiang Province, 310003, China

**Keywords:** Apigenin, Bladder cancer, Cell cycle arrest, Apoptosis, Invasion

## Abstract

**Background:**

Apigenin (4’,5,7-trihydroxyflavone) was recently shown effective in inhibiting several cancers. The aim of this study was to investigate the effect and mechanism of apigenin in the human bladder cancer cell line T24 for the first time.

**Methods:**

T24 cells were treated with varying concentrations and time of apigenin. Cell viability was evaluated by MTT assay. Cell motility and invasiveness were assayed by Matrigel migration and invasion assay. Flow cytometry and western blot analysis were used to detect cell apoptosis, cell cycle and signaling pathway.

**Results:**

The results demonstrated that apigenin suppressed proliferation and inhibited the migration and invasion potential of T24 bladder cancer cells in a dose- and time-dependent manner, which was associated with induced G2/M Phase cell cycle arrest and apoptosis. The mechanism of action is like to involve PI3K/Akt pathway and Bcl-2 family proteins. Apigenin increased caspase-3 activity and PARP cleavage, indicating that apigenin induced apoptosis in a caspase-dependent way.

**Conclusions:**

These findings suggest that apigenin may be an effective way for treating human bladder cancer.

## Background

Apigenin, one of the most common flavonoids, is widely distributed in many fruits and vegetables, including parsley, onions, orange, tea, chamomile, wheat sprouts and in some seasonings [1]. Apigenin has potential uses in cancer prevention and therapy, and it suppresses cell growth against many human cancer cell lines, including breast, colon, skin, thyroid, leukemia, and prostate cancer cells [2-5]. Unlike other structurally related flavonoids, apigenin is non-mutagenic [6]. Although previous reports have shown the inhibitory effect of apigenin on other human cancer cells, there are few reports indicating the inhibitory effect on human bladder cancer cells.

Bladder cancer is the most common malignant tumor of the urinary tract. Worldwide, bladder cancer is the seventh most common cancer. An average of 386,300 new cases of urinary bladder cancer are diagnosed worldwide every year, accounting for 150,200 deaths [7]. In recent decades, bladder cancer was shown of a rising overall incidence [8]. In most cases of nonmuscle invasive bladder cancer, tumors are treated initially with TURBT (Transurethral Resection of Bladder Tumor). A careful cystoscopic examination of the entire urethra and all bladder surfaces precedes resection. Intravesical therapy can also be employed in an expectant way as opposed to an induction course alone to provide long-term immunostimulation or chemotoxicity and thereby prevent disease recurrence [9,10]. Our earlier studies have shown that EGCG [11] and resveratrol [12] may be an important chemopreventive agent for the management of bladder cancer. Here we proved for the first time that apigenin could induce apoptosis and cell cycle arrest of bladder cells.

Besides many natural agents extracts like EGCG, resveratrol and genistein which are proved of the ability of cancer chemoprevention, apigenin is another agent we often contact. This study was designed to determine whether apigenin decreases the ability of migration and invasion of T24 bladder cells and is apoptotic of T24 bladder cells by inhibiting PI3K/Akt pathway, activating caspases and induces cell cycle arrest. Finally, we showed that in T24 bladder cancer cells apigenin upregulates Bax and Bad, activates caspase-3 and poly (ADPribose) polymerase (PARP), inhibits PI3K/Akt pathway, downregulates antiapoptotic protein Bcl-2 and Bcl-x, and leads to G2/M cell cycle arrest.

## Results

### Apigenin inhibits cell growth in T24 cells

**Figure 1:**
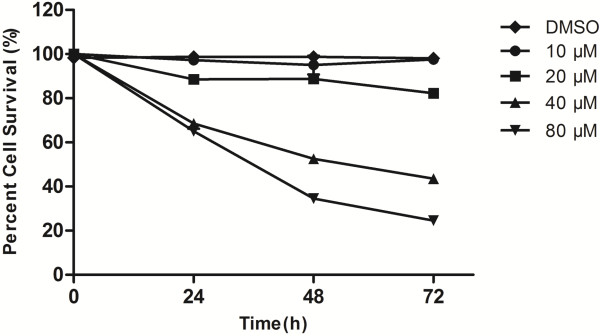
**Cytotoxicity effects of apigenin in T24 bladder cancer cells.** Cell proliferation and viability were determined by an MTT assay. Reduced cell viability with apigenin treatment (1 uL/mL DMSO, Apigenin of 10–80 μM) was observed at 24, 48 and 72 h. The data are presented as mean ± SD.

The MTT assay demonstrated that apigenin treatment with the vehicle DMSO (1 ul/mL) and varying concentrations (10–80 μM) and times (24 to 72 hours), resulted in a dose- and time-dependent inhibition of T24 cell growth, compared to untreated controls. As is shown in Figure 1, there was no significant difference between untreated control and vehicle control which meant DMSO wasn’t able to affect the proliferation of T24 cells. When the treated concentration was 10 μM, the viability of cells changed very little. Just because of this, we use the concentration of 0–20 μM to complete the migration and invasion assay. With the increasing of the concentration and time, there appeared an obvious reduction in cell viability, especially with the concentration of 40 and 80 μM. The inhibitory concentration 50% values for apigenin treatment were estimated to be 82.5, 52.9, and 43.8 μM for 24, 48, and 72 h, respectively. These data indicated that apigenin exerts a significant inhibitory effect on T24 cells.

### Apigenin inhibits T24 cell migration and invasion

**Figure 2:**
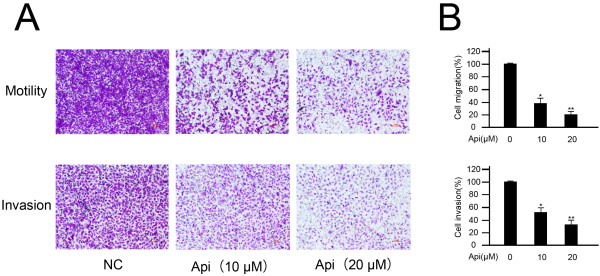
**Effect of apigenin on T24 cell invasion and migration.** Cells treated with 0–20 μM apigenin were induced to move or invade through uncoated or Matrigel-coated transwell membranes. After 24 hours, cells were fixed, stained, and counted. **A**, a representative image; **B**, shows the quantitation of one experiment done in triplicate. * *P* < 0.05 versus untreated control, ** *P* < 0.01 versus untreated control.

As the low concentration of apigenin didn’t induce a significant death of T24 cells, we treated the T24 cells with 0–20 μM to detect whether the low concentration of apigenin decreased the migration and invasion potential of T24 cells. As shown in Figure 2, apigenin-treated cells exhibited a significant decrease in both motility and invasion compared to untreated control.

### Apigenin induces apoptosis in T24 cells

**Figure 3:**
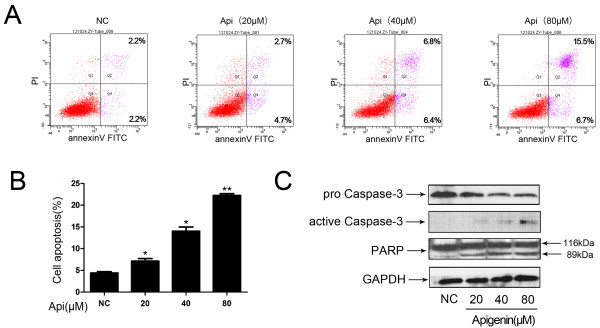
**Apigenin treatment induces dose-dependent apoptosis in T24 human bladder cancer cells.** (**A**) Dose-dependent apoptosis induced by treatment with apigenin in T24 bladder cancer cells. Cells treated with various concentrations of apigenin were double-stained with annexin V and PI and analyzed by flow cytometry. The gate setting distinguished between living (bottom left), necrotic (top left), early apoptotic (bottom right), and late apoptotic (top right) cells. (**B**) The percent of total apoptotic cells was quantified from three independent experiments. * *P* < 0.05, ** *P* < 0.01 versus untreated control. (**C**) Apigenin treatment activates caspase-3 and PARP in T24 human bladder cancer cells. The expression of proteins in treated cells was analyzed by Western blotting as detailed in Methods. A representative blot is shown from three independent experiments with identical results.

To determine whether the observed apigenin-induced cell death in T24 cells occurred via induction of apoptosis, we assessed the effect of apigenin treatment on apoptosis in the next series of experiments in T24 cells. The cells were treated with varying concentration of apigenin (0–80 μM) for 24 h and analyzed by flow cytometry. Compared with untreated control, apigenin treatment resulted in apoptosis in a dose-dependent manner (Figure 3A and B). The percent of apoptotic cells increased to 22.2% under the treatment of apigenin in the concentration of 80 μM.

Caspase-3 activation and PARP cleavage are characteristic indicators of apoptosis. As a downstream effector in the caspase cascade, when caspase-3 is activated, it induces an irreversible apoptosis [13]. PARP cleavage, procaspase-3 and active caspase-3 protein were detected by western blot. In apigenin-treated T24 cell samples, cleaved PARP and active caspase-3 increased, while procaspase-3 decreased in a dose-dependent manner after 24 h treatment (Figure 3C).

### Apigenin induces G2/M phase cell cycle arrest

**Figure 4:**
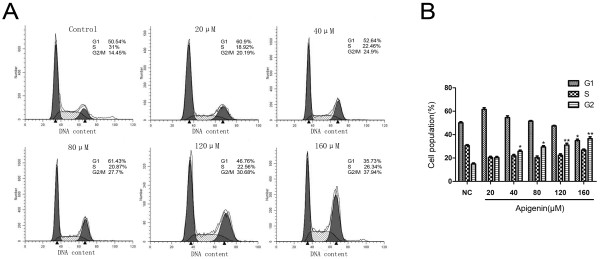
**Cell cycle analysis of T24 bladder cancer cells treated with apigenin.** (**A**)Cells were treated with different concentrations of apigenin for 24 h and then stained with propidium iodide. The DNA content was analyzed by flow cytometry. G1, S, and G2/M indicate cell cycle phase. (**B**) It shows an obvious G2/M phase arrest with a concomitant decrease of cell number in G1 phase of the cell cycle. The percent of G2/M phase was quantified from three independent experiments. * *P* < 0.05, ** *P* < 0.01 versus untreated control.

Treatment of T24 cells with apigenin resulted in dose- and time-dependent inhibition of cell growth and induced apoptosis, compared with their untreated controls. We considered the possibility that this may involve an arrest of cells at specific check point in the cell cycle. We therefore assessed the effect of apigenin on cell cycle perturbations. Compared with the untreated controls, apigenin treatment leaded to an appreciable arrest of T24 cells in G2/M phase of the cell cycle. Cell cycle analysis showed that the G2/M phase population of the control cells was 14.45% and the percentage of cells in G2/M phase significantly increased after 24 h treatment of apigenin of different concentrations. (Figure 4A) (20.19%, 24.9%, 27.7%, 30.68%, and 37.94% cells at 20, 40, 80, 120, and 160 μM concentrations of apigenin, respectively) after 24 h treatment. This increase in G2/M phase cell population was accompanied with a concomitant decrease of cell number in G1 phase of the cell cycle (Figure 4B).

### Apigenin modulates Akt pathway

**Figure 5:**
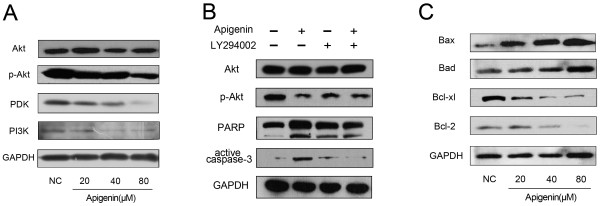
**Expression of proteins changed in cells treated with different concentrations of apigenin or apigenin and LY294002 for 24 h.** (**A**) Apigenin reduced the expression phosphorylation of Akt, PI3K and PDK significantly, but the expression of total Akt had no change. (**B**) T24 cells was treated with 20 μM PI3 kinase inhibitor LY294002 for 30 min and then with 40 μM apigenin for another 24 h. PI3K inhibitor, LY294002 decreased the protein levels of cleaved PARP and active casepase-3 which were activated by apigenin. (**C**) Apigenin inhibited the expression of anti-apoptotic gene products Bcl-2 and Bcl-xL but upregulated the expression of apoptotic gene product Bax and Bad. A representative blot is shown from three independent experiments with identical results.

Akt acts as an anti-apoptotic signaling molecule and is a good candidate for mediating the PI3K-dependent cell-survival responses [14]. To determine whether apigenin induces apoptosis by modulation of this pathway, we investigated the expression of total Akt and phosphorylation of Akt after treatment with apigenin of different concentrations (0–80 μM) for 24 h. Western blot analysis showed that the expression of phosphorylation of Akt (p-Akt) is decreased in a dose-dependent way, while no significant difference existed in total Akt (Figure 5A). At the same time, the protein PI3K and PDK, which phosphorylates Akt, also showed a decrease. These results indicate PI3K/Akt pathway plays an important role in the apigenin-induced apoptosis in T24 bladder cancer.

To further investigate the modulation of apigenin on PI3K-Akt pathway, T24 cells were pretreated with 20 μM PI3 kinase inhibitor LY294002 for 30 min. Cells were than treated with 40 μM apigenin for another 24 h. Cellular proteins were extracted and analyzed by Western blotting. As shown in Figure 5B, PI3K inhibitor, LY294002 decreased the protein levels of cleaved PARP and active casepase-3, suggesting that apigenin-induced apoptosis depended on PI3K-Akt activity.

### Apigenin alters Bcl-2 family protein expression in T24 cells

As is known, Bcl-2 family plays a crucial role in apoptosis. The change of the ratio of proapoptotic protein versus antiapoptotic proteins of Bcl-2 family such as Bax and Bcl-2 will activate the mitochondrial apoptotic pathway. In addition, several kinases have been shown to phorylate and inactivate Bad, and Akt is one of them [15]. Therefore we next studied the dose-dependent effects of apigenin on the constitutive protein levels of Bcl-2 family in T24 cells. The Western blot analysis showed a significant increase in the expression of pro-apoptotic protein Bax and Bad, while in sharp contrast, the protein expression of Bcl-2 and Bcl-xl was significantly decreased by apigenin treatment in a dose-dependent manner (Figure 5C). The results revealed evidence that apigenin-induced apoptosis was involved with Bcl-2 family.

## Discussion

In this study, we showed that apigenin, a nonmutagenic antitumor flavonoid, exhibits an inhibition action on T24 bladder cancer cells for the first time. We confirmed the chemopreventive/therapeutic potential of apigenin against bladder cancer by induction of apoptosis, migration and invasion inhibition and cell cycle arrest. Akt, also known as Protein Kinase B (PKB), is a serine/threonine-specific protein kinase that plays a key role in multiple cellular processes such as glucose metabolism, apoptosis, cell proliferation, transcription and cell migration. The mechanism by which Akt protects cells from death is likely to be multifactorial, because Akt directly phosphorylates several components of the cell-death machinery [16]. Some of the mechanisms involve the phosphorylation and inactivation of the apoptotic mediators Bad, caspase-9, FKHRL1, and IKK [17-23]. Besides, Akt is known to be a downstream of PI3K to regulate many biological processes. In our study, we confirmed that apigenin treatment in T24 cells induced apoptosis and inhibited the phosphorylation of Akt in a dose-dependent manner which meant the apigenin-treated apoptosis was involved with PI3K/Akt pathway. Notably, our data suggest that the mechanism of the tumor suppressive effect involved inhibition of PI3K/Akt signaling pathways. In this study, the reason why the PI3K/Akt expression level was markedly decreased by apigenin treatment is not clear. An earlier study on head and neck carcinomas suggests that apigenin targets EGFR, which is upstream of PI3K/Akt [24]. In a future study, the correlation between apigenin and EGFR expression levels in bladder cancer needs to be examined.

The Bcl-2 protein family consists of both pro-apoptotic (Bax and Bad) and anti-apoptotic (Bcl-2) proteins that regulate mitochondrial outer membrane integrity, cytochrome *c* release, and caspase activation leading to apoptosis. Previous studies showed that with the activation of the PI3K/Akt pathway the expression of Bcl-2 family increased [25], and Akt inhibits apoptosis through mitochondrial pathways [26]. Shifting the balance of Bcl-2 family members toward pro-apoptotic effects will activate caspase-3 and executes the apoptotic program [27]. Therefore we investigated the effect of apigenin on Bcl-2 family. The present study indicates that apigenin treatment upregulates pro-apoptotic proteins Bax and Bad while downregulates anti-apoptotic proteins Bcl-2 and Bcl-xl protein. Change of the Bcl-2 family induces the release of cytochrome c from mitochondria into cytosol and cytosolic cytochrome c then binds to Apaf-1 and leads to the activation of caspase-3 and PARP [28]. In our research, we also confirmed that apigenin activated caspase-3 and leaded to PARP cleavage. Thus our study proved apigenin treatment induces apoptosis in T24 cells via PI3K/Akt pathway and Bcl-2 family.

Cell cycle arrest and apoptosis represent two effective mechanisms involved in the induction of cell death [29]. It is well established that loss of key cell cycle checkpoints is a hallmark of cancer cells, leading to abnormal proliferation and facilitating oncogenic transformation [30]. Observations have shown that apigenin is a potent inhibitor of cell-cycle progression in a number of different cell lines [31,32]. We also measured the effect of apigenin on cell cycle of T24 cells and found that apigenin leads to a G2/M phase arrest. The similar results were observed in human colon and breast carcinomas [4]. In the present study, G2/M phase increased from 14.45% up to 37.94%, with almost 2.6 folds increasing, in a dose-dependent way, which indicated the apigenin-induced cell growth inhibition was involved with cell cycle arrest. Although Lepley DM, et al. [32] have proved a G1 arrest by apigenin in human diploid fibroblast, we observed G2/M arrest in apigenin-treated T24 cells. The difference between these results might be attributed to the cell types tested. Previous studies have shown that PI3K/Akt pathway could regulate expression of G2/M-related proteins to influence the progression of G2 to mitosis phase. Expression of active form of Akt led to an increase in the protein and mRNA level of Cdk1, whereas Akt dominant negative mutation inhibited cell proliferation by inducing G2/M arrest [33]. Taken together, apigenin may inhibit cellular proliferation by inducing a cell cycle arrest at G2/M in T24 bladder cancer cells and probably via PI3K/Akt pathway.

## Conclusion

In conclusion, our study demonstrates that apigenin can induce dose- and time-dependent cell death and apoptosis and inhibit migration and invasion ability in T24 bladder cancer cells. Apigenin leads to apoptosis via PI3K/Akt pathway, regulation of Bcl-2 family and activation of caspase-3 and PARP. Additionally, Apigenin also causes G2/M phase arrest. All these results indicate that apigenin can be used as a chemopreventive agent in bladder cancer. To the best of our knowledge, this is the first report showing the antitumor effect of apigenin in bladder cancer in vitro. However, further investigations of the mechanism of apigenin-treated cell inhibition are necessary.

## Methods

### Reagents and cell culture

Apigenin (≥ 99% pure) and MTT were purchased from Sigma Chemical Co. (St. Louis, MO, USA). The annexin V-FITC apoptosis detection kit was from BD Biosciences (SanJose, CA, USA). Primary antibodies to Bcl-2, Bax, Bcl-xL, pro caspase-3, active caspase-3, GAPDH and poly(ADP-ribose) polymerase (PARP), and secondary antibodies were purchased from Santa-Cruz Biotechnology, Inc. (Santa Cruz, CA). Antibodies to Akt, phosphorylated Akt, PDK, PI3K and Bad were purchased from Cell Signaling Technology (Beverly, MA). The bicinchoninic acid protein assay kit was obtained from Pierce Biotechnology (Rockford, IL).

The human bladder cancer cell line T24 was obtained from the Shanghai Institute of Cell Biology, Chinese Academy of Sciences (Shanghai, China). The cells were cultured in RPMI-1640 medium (HyClone, Logan, UT, USA) supplemented with 10% heat-inactivated FBS (JRH Biosciences, Lenexa, KS, USA), 100 U/ml penicillin, and 100 mg/L strep-tomycin. Cultures were maintained in a humidified atmosphere of 5% CO_2_ at 37°C.

### Cell viability assay

The effect of apigenin on the viability of T24 cells was evaluated by MTT assay. Approximately 10 × 10^4^ T24 cells were seeded on 96-well plates. After overnight incubation, the cells were treated with vehicle DMSO (1 uL/mL) and different concentrations of apigenin (0–80 μM in DMSO) for 24 hours. After incubation for the indicated time, MTT (20 μL of 5 mg/mL) was added to each well and incubated at 37°C for 4 h, after which the MTT solution in the medium was removed. To achieve solubilization of the formazan crystal formed in viable cells, 150 μL DMSO was added to each well before the absorbance at 490 nm was measured using an MRX II absorbance reader (Dynex Technologies, Chantilly, VA, USA). Results were expressed as a percentage of growth, with 100% representing control cells treated with DMSO alone.

### In vitro invasion and motility assays

The invasion and motility assays were done as previously described [34] with some minor modifications. Cells were plated in a 6-well plate at a density of 8 × 10^4^ cells/well. After overnight incubation, cells were treated with different concentrations of apigenin (0–20 μM in DMSO) for 24 hours and harvested. The treated and the control cells were suspended in medium at a concentration of 4 × 10^5^ cells/mL, and 0.2 mL of each was added to the top chamber of uncoated (for motility assays) or Matrigel-coated (for invasion assays) PET membranes (24-well insert, 8-μm pore size; Millipore, Bedford, MA). Medium (0.6 mL) supplemented with 20% fetal bovine serum was added to each well of the plate to act as a chemoattractant in the lower chamber. Cells were incubated for 24 hours, and those that did not migrate through the pores were removed by scraping the upper surface of the membrane with a cotton swab. Cells that had migrated to the lower surface of the membrane were fixed for 5 min in 100% methanol and stained with 0.1% crystal violet for 2 min. These experiments were done in triplicate and performed a minimum of three times.

### Cell apoptosis assay

The extent of apoptosis was evaluated by annexin V-FITC and flow cytometry. Cells were grown at a density of 1 × 10^6^ cells in six-well culture dishes and were treated with different concentrations of apigenin (0–80 μM in DMSO) for 24 h. Following treatment, the cells were harvested, washed twice with pre-chilled PBS, and resuspended in 1× binding buffer at a concentration of 1 × 10^6^ cells/ml. One hundred microliters of such solution was mixed with 5 μL annexin V-FITC and 5 μL propidium iodide for 15 min, and then 400 μL 1× binding buffer was added. Analysis was carried out using a FC500 flow cytometer with CXP software (Beckman Coulter, Fullerton, CA, USA) within 1 h. The percentage of apoptotic cells was assessed by CXP software.

### Cell cycle assay

Cells were plated in six-well culture dishes at concentrations determined to yield 60–70% confluence within 24 h. Cells were then treated with different concentrations of apigenin (0–160 μM in DMSO). After 24 h, cells were washed twice with PBS then centrifuged. The pellet was fixed with 70% ethanol for 1 h at 4°C. The cells were washed with PBS and resuspended with propidium iodide solution (0.05 mg/mL) containing RNase, incubated at room temperature in the dark for 30 min. DNA content was then analyzed using the FC500 flow cytometer.

### Western blot analysis

Cell were harvested at 24 h following apigenin treatment, washed, and lysed with lysis buffer (10 mmol/L Tris-HCl, 0.25 mol/ L sucrose, 5 mmol/L EDTA, 50 mmol/L NaCl, 30 mmol/L sodium pyrophosphate, 50 mmol/L NaF, 1 mmol/LNa3VO4, 1 mmol/L PMSF, and 2% cocktail [pH 7.5]). Protein concentration in the resulting lysate was determined using the bicinchoninic acid protein assay. Appropriate amounts of protein (20–30 μg) were separated by electrophoresis in 10–12% Tris-glycine polyacrylamide gels and transferred to nitrocellulose membranes. Membranes were blocked then incubated overnight with the appropriate primary antibody at dilutions specified by the manufacturer. They were next washed and incubated with the corresponding HRP-conjugated secondary antibody at 1:1000 dilution in Tris-buffered saline-Tween 20 (10 mM Tris-Cl [pH 7.4], 150 mM NaCl, 0.1% Tween-20). Bound secondary antibody was detected using an enhanced chemiluminescence system (Pierce Biotechnology).

### Statistical analysis

Statistical significance was compared between various treatment groups and controls using ANOVA. Data were considered statistically significant when P-values were <0.05.

## Competing interests

The authors declare that they have no competing interests.

## Authors’ contributions

YZ participated in all aspects of the experiment and the article. YM, HC and YL carried out the molecular studies and drafted the manuscript. XiaX, XX, JW and ZH participated in the assays. JQ participated in the design of the study and performed the statistical analysis. LX conceived of the study, and participated in its design and coordination and helped to draft the manuscript. All authors read and approved the final manuscript.

## References

[B1] Birt DF Mitchell D Gold B Pour P Pinch HC Inhibition of ultraviolet light induced skin carcinogenesis in SKH-1 mice by apigenin, a plant flavonoid Anticancer Res 1997 17 1A 85 91 9066634

[B2] Caltagirone S Rossi C Poggi A Ranelletti FO Natali PG Brunetti M Aiello FB Piantelli M Flavonoids apigenin and quercetin inhibit melanoma growth and metastatic potential Int J Cancer 2000 87 4 595 600 10.1002/1097-0215(20000815)87:4<595::AID-IJC21>3.0.CO;2-5 10918203

[B3] Wang IK Lin-Shiau SY Lin JK Induction of apoptosis by apigenin and related flavonoids through cytochrome c release and activation of caspase-9 and caspase-3 in leukaemia HL-60 cells Eur J Cancer 1999 35 10 1517 1525 10.1016/S0959-8049(99)00168-9 10673981

[B4] Wang W Heideman L Chung CS Pelling JC Koehler KJ Birt DF Cell-cycle arrest at G2/M and growth inhibition by apigenin in human colon carcinoma cell lines Mol Carcinog 2000 28 2 102 110 10.1002/1098-2744(200006)28:2<102::AID-MC6>3.0.CO;2-2 10900467

[B5] Yin F Giuliano AE Law RE Van Herle AJ Apigenin inhibits growth and induces G2/M arrest by modulating cyclin-CDK regulators and ERK MAP kinase activation in breast carcinoma cells Anticancer Res 2001 21 1A 413 420 11299771

[B6] Czeczot H Tudek B Kusztelak J Szymczyk T Dobrowolska B Glinkowska G Malinowski J Strzelecka H Isolation and studies of the mutagenic activity in the Ames test of flavonoids naturally occurring in medical herbs Mutat Res 1990 240 3 209 216 10.1016/0165-1218(90)90060-F 2179716

[B7] Jemal ABF Center MM Ferlay J Ward E Forman D Global cancer statistics CA Cancer J Clin 2011 61 69 90 10.3322/caac.20107 21296855

[B8] Weir HK Thun MJ Hankey BF Ries LA Howe HL Wingo PA Jemal A Ward E Anderson RN Edwards BK Annual report to the nation on the status of cancer, 1975–2000, featuring the uses of surveillance data for cancer prevention and control J Natl Cancer Inst 2003 95 17 1276 1299 10.1093/jnci/djg040 12953083

[B9] De Boer EC De Jong WH Steerenberg PA Aarden LA Tetteroo E De Groot ER Van der Meijden AP Vegt PD Debruyne FM Ruitenberg EJ Induction of urinary interleukin-1 (IL-1), IL-2, IL-6, and tumour necrosis factor during intravesical immunotherapy with bacillus Calmette-Guerin in superficial bladder cancer Cancer Immunol Immunother 1992 34 5 306 312 10.1007/BF01741551 1540977 PMC11038144

[B10] Lamm DL Blumenstein BA Crissman JD Montie JE Gottesman JE Lowe BA Sarosdy MF Bohl RD Grossman HB Beck TM Maintenance bacillus Calmette-Guerin immunotherapy for recurrent TA, T1 and carcinoma in situ transitional cell carcinoma of the bladder: a randomized Southwest Oncology Group Study J Urol 2000 163 4 1124 1129 10.1016/S0022-5347(05)67707-5 10737480

[B11] Qin J Xie LP Zheng XY Wang YB Bai Y Shen HF Li LC Dahiya R A component of green tea, (-)-epigallocatechin-3-gallate, promotes apoptosis in T24 human bladder cancer cells via modulation of the PI3K/Akt pathway and Bcl-2 family proteins Biochem Biophys Res Commun 2007 354 4 852 857 10.1016/j.bbrc.2007.01.003 17266926

[B12] Bai Y Mao QQ Qin J Zheng XY Wang YB Yang K Shen HF Xie LP Resveratrol induces apoptosis and cell cycle arrest of human T24 bladder cancer cells in vitro and inhibits tumor growth in vivo Cancer Sci 2010 101 2 488 493 10.1111/j.1349-7006.2009.01415.x 20028382 PMC11159480

[B13] Boatright KM Salvesen GS Mechanisms of caspase activation Curr Opin Cell Biol 2003 15 6 725 731 10.1016/j.ceb.2003.10.009 14644197

[B14] Franke TF Hornik CP Segev L Shostak GA Sugimoto C PI3K/Akt and apoptosis: size matters Oncogene 2003 22 56 8983 8998 10.1038/sj.onc.1207115 14663477

[B15] Pugazhenthi S Nesterova A Sable C Heidenreich KA Boxer LM Heasley LE Reusch JE Akt/protein kinase B up-regulates Bcl-2 expression through cAMP-response element-binding protein J Biol Chem 2000 275 15 10761 10766 10.1074/jbc.275.15.10761 10753867

[B16] Vivanco I Sawyers CL The phosphatidylinositol 3-Kinase AKT pathway in human cancer Nat Rev Cancer 2002 2 7 489 501 10.1038/nrc839 12094235

[B17] Datta SR Dudek H Tao X Masters S Fu H Gotoh Y Greenberg ME Akt phosphorylation of BAD couples survival signals to the cell-intrinsic death machinery Cell 1997 91 2 231 241 10.1016/S0092-8674(00)80405-5 9346240

[B18] Cardone MH Roy N Stennicke HR Salvesen GS Franke TF Stanbridge E Frisch S Reed JC Regulation of cell death protease caspase-9 by phosphorylation Science 1998 282 5392 1318 1321 9812896 10.1126/science.282.5392.1318

[B19] Brunet A Bonni A Zigmond MJ Lin MZ Juo P Hu LS Anderson MJ Arden KC Blenis J Greenberg ME Akt promotes cell survival by phosphorylating and inhibiting a Forkhead transcription factor Cell 1999 96 6 857 868 10.1016/S0092-8674(00)80595-4 10102273

[B20] Ozes ON Mayo LD Gustin JA Pfeffer SR Pfeffer LM Donner DB NF-kappaB activation by tumour necrosis factor requires the Akt serine-threonine kinase Nature 1999 401 6748 82 85 10.1038/43466 10485710

[B21] Romashkova JA Makarov SS NF-kappaB is a target of AKT in anti-apoptotic PDGF signalling Nature 1999 401 6748 86 90 10.1038/43474 10485711

[B22] Ahmed NN Grimes HL Bellacosa A Chan TO Tsichlis PN Transduction of interleukin-2 antiapoptotic and proliferative signals via Akt protein kinase Proc Natl Acad Sci U S A 1997 94 8 3627 3632 10.1073/pnas.94.8.3627 9108028 PMC20491

[B23] Medema RH Kops GJ Bos JL Burgering BM AFX-like Forkhead transcription factors mediate cell-cycle regulation by Ras and PKB through p27kip1 Nature 2000 404 6779 782 787 10.1038/35008115 10783894

[B24] Masuelli L Marzocchella L Quaranta A Palumbo C Pompa G Izzi V Canini A Modesti A Galvano F Bei R Apigenin induces apoptosis and impairs head and neck carcinomas EGFR/ErbB2 signaling Front Biosci 2011 16 1060 1068 10.2741/3735 21196218

[B25] Asnaghi L Calastretti A Bevilacqua A D'Agnano I Gatti G Canti G Delia D Capaccioli S Nicolin A Bcl-2 phosphorylation and apoptosis activated by damaged microtubules require mTOR and are regulated by Akt Oncogene 2004 23 34 5781 5791 10.1038/sj.onc.1207698 15208671

[B26] Chan TO Rittenhouse SE Tsichlis PN AKT/PKB and other D3 phosphoinositide-regulated kinases: kinase activation by phosphoinositide-dependent phosphorylation Annu Rev Biochem 1999 68 965 1014 10.1146/annurev.biochem.68.1.965 10872470

[B27] Brown GC Borutaite V Nitric oxide, cytochrome c and mitochondria Biochem Soc Symp 1999 66 17 25 10989653 10.1042/bss0660017

[B28] Yang J Liu X Bhalla K Kim CN Ibrado AM Cai J Peng TI Jones DP Wang X Prevention of apoptosis by Bcl-2: release of cytochrome c from mitochondria blocked Science 1997 275 5303 1129 1132 10.1126/science.275.5303.1129 9027314

[B29] King KL Cidlowski JA Cell cycle regulation and apoptosis Annu Rev Physiol 1998 60 601 617 10.1146/annurev.physiol.60.1.601 9558478

[B30] Pan J She M Xu ZX Sun L Yeung SC Farnesyltransferase inhibitors induce DNA damage via reactive oxygen species in human cancer cells Cancer Res 2005 65 9 3671 3681 10.1158/0008-5472.CAN-04-2744 15867362

[B31] Lepley DM Li B Birt DF Pelling JC The chemopreventive flavonoid apigenin induces G2/M arrest in keratinocytes Carcinogenesis 1996 17 11 2367 2375 10.1093/carcin/17.11.2367 8968050

[B32] Lepley DM Pelling JC Induction of p21/WAF1 and G1 cell-cycle arrest by the chemopreventive agent apigenin Mol Carcinog 1997 19 2 74 82 10.1002/(SICI)1098-2744(199707)19:2<74::AID-MC2>3.0.CO;2-L 9210954

[B33] Lee SRPJ Park EK Chung CH Kang SS Bang OS Akt-induced promotion of cell-cycle progression at G2/M phase involves upregulation of NF-Y binding activity in PC12 cells J Cell Physiol 2005 205 2 270 277 10.1002/jcp.20395 15887249

[B34] Albini A Iwamoto Y Kleinman HK Martin GR Aaronson SA Kozlowski JM McEwan RN A rapid in vitro assay for quantitating the invasive potential of tumor cells Cancer Res 1987 47 12 3239 3245 2438036

